# Leaky by Design: Unlocking Polymersome Permeability Using Moderately Hydrophobic Polymer Blocks

**DOI:** 10.1021/acs.langmuir.5c06389

**Published:** 2026-03-11

**Authors:** Wencui Zhang, Anabella P. Rosso, Yang Yu, Fernando Augusto de Oliveira, Martina Vragovic, Cécile Huin, Philippe Guégan, Guillaume Tresset, Fernando Carlos Giacomelli

**Affiliations:** 1 Equipe Chimie des Polymères, Institut Parisien de Chimie Moléculaire (UMR-CNRS 8232), Sorbonne Université, Paris 75 252, France; 2 Centro de Ciências Naturais e Humanas, Universidade Federal do ABC, Santo André 09210-580, Brazil; 3 Institute of Macromolecular Chemistry, Czech Academy of Sciences, Prague 162 00, Czech Republic; 4 Université Evry Paris-Saclay, Evry 91 000, France; 5 Université Paris-Saclay, CNRS, Laboratoire de Physique des Solides, Orsay 91 405, France

## Abstract

Polymersome nanoreactors, vesicular assemblies formed from amphiphilic block copolymers, provide a versatile platform for compartmentalized catalysis and the construction of biomimetic systems. While extensive efforts have focused on the encapsulation of enzymes within such constructs, reports of vesicles displaying intrinsic membrane permeability remain unusual. Typically, selective transport across polymersome membranes requires the incorporation of channel proteins or other porogenic components. Conversely, we herein demonstrate that polymer vesicles comprising poly­(butylene oxide) as the hydrophobic segment exhibit inherent permeability to small molecules without the need for artificial machineries, possibly governed by moderate hydrophobicity of this polymer and consequently hydration of the membrane. Synthesis and detailed characterization of diblock and triblock copolymers containing poly­(butylene oxide) and poly­(glycidol), respectively, as hydrophobic and hydrophilic blocks are first demonstrated; the self-assembly of the chains into polymer vesicles and their inherent permeability to disparate small molecules are subsequently highlighted. Notably, we further reveal that such vesicles can be conveniently loaded with a model enzyme (horseradish peroxidase), which remains entrapped in the aqueous lumen. Using a well-established colorimetric assay, we show that the vesicles are also permeable to small-enzyme substrates and products, and therefore, the reported strategy can be applied to a wide range of enzymes and functional proteins for the design of simple permeable nanoreactors for enzyme-mediated catalysis.

## Graphical abstract

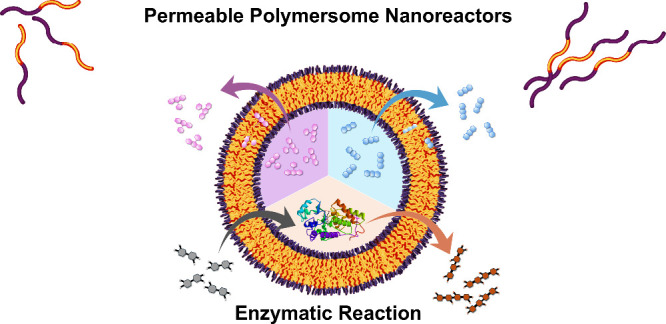

## Introduction

Polymersomes are vesicular assemblies composed of an aqueous lumen enclosed by a polymeric bilayer membrane. Owing to their structural versatility, these compartmentalized nanostructures have been widely explored as platforms for artificial cells and organelles,^1,2^ catalytic nanoreactors (nanofactories),^3–5^ and advanced devices for biomedical applications.^6,7^ Such applications are linked to the compartmentalization provided by the constructs. While the confined environments allow the protection of biomacromolecules from detrimental agents, the communication between the inner and outer compartments and the capability of the self-assemblies to regulate the flow of chemicals are of due relevance, and it truly guides numerous biorelated potential applications.^8,9^ Compared to liposomes, owing to their thicker and mechanically robust membranes formed by high-molecular-weight molecules, polymersomes are generally more stable. On the other hand, polymer vesicles typically display poor membrane permeability due to low lateral diffusivity and high bending rigidity.^10^ Nevertheless, for most of the applications, the incoming and outgoing of molecules across polymeric walls are needed at least to a certain extent. Cargo delivery systems are expected to protect therapeutic agents during systemic circulation while the release at a target site is the main task.^11,12^ Polymersome-based catalytic nanoreactors rely on controlled membrane permeability, enabling the diffusion of low-molecular-weight substrates and products while confining enzymes within the aqueous core.^13,14^ Permeability is also needed in the mimicking of cell-like organelles since most of the processes are triggered by passive diffusion of molecules and ions across membranes.^15,16^

The permeability of polymersomes is primarily governed by the structural features and chemical nature of the constituents, and low membrane permeability is a common feature.^17,18^ This constraint is often overcome through the incorporation of transmembrane proteins^19,20^ and DNA nanopores,^21,22^ thus making nanochannels toward size-dependent polymersome permeability. Conversely, the fabrication of intrinsically permeable or semipermeable polymer membranes without the use of external porogens or additives remains scarcely reported, with only a limited number of polymer vesicles known to exhibit inherent permeability to date. One can cite polystyrene-*b*-poly­(isocyanoalanine­(2-thiophen-3-yl-ethyl)­amide), which has been proven to be inherently porous due to poor polymer packing.^23^ Poly­(ethylene glycol)-*b*-poly­(2-hydroxypropyl methacrylate) polymersomes have likewise been reported to display intrinsic permeability, attributed to the hydrated character of the moderately hydrophobic poly­(2-hydroxypropyl methacrylate) membrane-forming segment.^24,25^ Permeable polymersomes have also been generated from poly­(propylene oxide)-based (PPO) copolymers due to the relatively high degree of hydration of the hydrophobic domain.^26^ Although the hydration of PBO membranes is reduced compared with PPO,^27^ it may explain the permeability of the PBO-based polymersomes produced by Battaglia et al.^28^ Notably, however, the same authors emphasized that these vesicles can remain impermeable, a finding corroborated by others,^29,30^ underscoring the key influence of the chemical nature of the probe on permeability behavior. Truthfully, the interplay between hydrophilicity and hydrophobicity in polymeric membranes plays a pivotal role in determining the permeability of polymersomes. Markedly, reactions involving highly hydrophobic polymers and the hydrophilic molecule 2-hydroxy-4′-2-(hydroxyethoxy)-2-methylpropiophenone (PP–OH) have been found to increase membrane permeability,^31^ thus allowing the production of nanofactories without the need of insertional nanopores. Similarly, the intrinsic permeability of polymer vesicles based on amino-containing polymers has been recently highlighted by us,^18,32^ thus enabling their use in the construction of catalytic microreactors.^14^ Undoubtedly, intrinsically permeable polymer membranes eliminate the need for multistep fabrication and postassembly pore formation, enabling the construction of highly versatile nanoreactor platforms.

Taking into account all of the above-mentioned considerations, here, we take steps forward by investigating block copolymers made by PBO as hydrophobic segment with different architectures, focusing on the development of intrinsically permeable PBO-based polymeric nanoreactors. With this in mind, we initially identified that PBO-based vesicles are inherently permeable to model fluorescent probes such as methylene blue (MB) and rhodamine B (RhB) possibly due to the highly amorphous feature of the membranes, low PBO glass transition temperature, and moderate hydrophobicity of the polymer block. We have further manufactured nanoreactors by entrapping horseradish peroxidase (HRP) in the hollow spheres, subsequently evaluating the oxidation of o-dianisidine to a red-brown dimer product detected by a colorimetric assay. The efficient HRP-catalyzed reaction in the presence of H_2_O_2_ underscores the potential of PBO-based intrinsically permeable nanoreactors, which enable versatile protein encapsulation for the synthesis of biologically relevant compounds, without requiring elaborate or costly membrane permeabilization strategies. These investigations accordingly sum up to a cohesive body of work, with each of them contributing to advances in the field.

## Materials and Methods

### Chemicals

Butylene oxide (BO, 99%, Sigma-Aldrich) and ethoxyethyl glycidyl ether (EEGE, prepared from glycidol, 96%, Sigma-Aldrich and ethyl vinyl ether, 99%, Sigma-Aldrich) were purified by two successive vacuum distillations over calcium hydride (CaH_2_, Sigma-Aldrich) and subsequently transferred into a glovebox for storage. Benzyl alcohol (BA, anhydrous, 99.8%, Sigma-Aldrich) and the phosphazene base tBuP_4_ (0.8 mol L^–1^ in *n*-hexane, Sigma-Aldrich) were also stored under an argon atmosphere inside a glovebox until use. Calcium hydride, neutral alumina (Sigma-Aldrich), 1,4-benzenedimethanol (BDM, 99%, Sigma-Aldrich), dichloromethane (DCM, VWR Chemicals), and methanol (MeOH, VWR Chemicals) were used as received. Toluene and tetrahydrofuran (THF) were purified using an MBRAUN MB SPS COMPACT solvent purification system equipped with alumina and copper catalyst columns. Polymerizations were performed in an MBRAUN stainless-steel glovebox filled with dry argon (H_2_O less than 0.5 ppm), where the moisture level was continuously monitored with an MB-MO-SE 1 probe. Horseradish peroxidase (HRP, Type II, lyophilized powder, 150–250 units per mg), o-dianisidine, rhodamine B (RhB), and methylene blue (MB) were also purchased from Sigma-Aldrich

### Synthesis of the Block Copolymers

The block copolymers were obtained via sequential anionic ring-opening polymerization. Diblock copolymer PGL_15_-*b*-PBO_44_ was synthesized as follows: initially, butylene oxide (BO, 576.9 mg, 8.0 mmol, 40 equiv) and a solution of tBuP_4_ (0.8 mol L^–1^ in *n*-hexane; 125 μL, 0.1 mmol, 0.5 equiv) were sequentially added to benzyl alcohol (BA, 20.8 μL, 0.2 mmol, 1 equiv) in tetrahydrofuran (THF, 0.35 mL) to initiate the homopolymerization of BO. As for PGL_26_-*b*-PBO_78_-*b*-PGL_26_, a solution of 1,4-benzenedimethanol (BDM, 20.7 mg, 0.15 mmol, 1 equiv) and BO (2.0 mL, 12.6 mmol, 84 equiv) in THF (1.3 mL) was reacted in the presence of tBuP_4_ (94 μL, 0.075 mmol, 0.5 equiv). Both chemical reactions were carried out at 50 °C for 24 h until complete consumption of BO as confirmed by ^1^H NMR. Subsequently, the second monomer EEGE (653.2 mg, 4.6 mmol, 23 equiv) has been added for the diblock synthesis, and the polymerization of EEGE was carried out at 50 °C for another 24 h. The same procedure was conducted for the triblock copolymer with a different EEGE amount (1.0 mL, 6.3 mmol, 42 equiv). ^1^H NMR spectroscopy was again employed to confirm the complete conversion of EEGE. Deionized water has been then added to quench the polymerizations and get the crude products, which were diluted with DCM and purified through a short column of neutral alumina to remove the catalyst (tBuP_4_). The eluted solutions were subsequently concentrated, yielding the diblock copolymer PEEGE_21_-*b*-PBO_43_ and the triblock copolymer PEEGE_24_-*b*-PBO_80_-*b*-PEEGE_24_ (as determined by ^1^H NMR analysis). The intermediate copolymers were then subjected to acidic treatment to deprotect the hydroxyl groups and reveal the hydrophilic functionality of polyglycidol (PGL) block. The polymers (*M*_n,theo_ = 6.3 kg mol^–1^; 0.63 g, 0.1 mmol for the diblock copolymer and *M*_n,theo_ = 9.3 kg mol^–1^, 0.93 g, 0.1 mmol for the triblock copolymer) were dissolved in MeOH and treated with HCl in MeOH (2 mol L^–1^, 2.4 mL, 4.2 mmol) at room temperature for 4 h. The reactions were then neutralized using sodium bicarbonate (NaHCO_3_), and the resulting byproducts were removed by filtration. The materials were further purified by dialysis against MeOH using regenerated cellulose membranes with a molecular weight cutoff (MWCO) of 1 kDa yielding hydroxyl-terminated purified PGL_15_-*b*-PBO_44_ and PGL_26_-*b*-PBO_78_-*b*-PGL_26_ block copolymers as white solids.

### Characterization of the Block Copolymers

^1^H NMR spectra were acquired on a Bruker Ultra Shield 400 MHz spectrometer, and chemical shifts were referenced to residual solvent peaks (CDCl_3_-d_1_ or MeOD-*d*_4_). The spectra were processed by using MestReNova 14.2.1. Size exclusion chromatography (SEC) measurements were performed in THF at 40 °C on three PL gel mixed-C 5 μm columns (7.5 × 300 mm; separation limits: 0.2 to 2000 kg mol^–1^), with a flow rate of 1.0 mL min^–1^, and using a sample Viscotek GPCmax delivery module and two modular detectors: a Viscoteck 3580 differential RI detector and a Shimadzu SPD20-AV diode array UV detector. Samples (5 mg mL^–1^ in THF with toluene as the marker) were filtered through 0.45 μm syringe filters prior to injection. Number-average molecular weights (*M*_n,GPC_) and dispersity indexes (*Đ*) were determined relative to PMMA standards (Polymer Standards Service) using RI detection, and data were analyzed using the OmniSEC 5.12 software.

### Preparation of the Probe-Loaded Polymersomes

Rhodamine B (RhB)- and methylene blue (MB)-loaded polymersomes were prepared by first dissolving 16 mg of each copolymer in 1.0 mL of methanol. An aqueous solution of RhB or MB (1.0 mg·mL^–1^ in phosphate buffer, PB) was then added dropwise to the 1.0 mL copolymer solution using a syringe pump (New Era Pump Systems, Inc.). A total of 2.0 mL of dye solution was introduced over 2 h: 0.5 mL during the first hour at 0.5 mL h^–1^, followed by 1.5 at 1.0 mL h^–1^. Subsequently, 0.5 mL of PBS was added at the same rate, and an additional 0.5 mL of PB was introduced in a single portion. The organic solvent was removed by slow evaporation, and the resulting dispersions were purified by G-50 column chromatography using PB (0.01 mol L^–1^, pH 7.4) as the eluent.

### Preparation of the Enzyme-Loaded Polymersomes

Copolymers (16 mg) were dissolved in methanol (1.0 mL), followed by the gradual addition of an aqueous HRP solution (50 μg mL^–1^, Milli-Q water) by using a syringe pump. A total of 2.5 mL was introduced in a stepwise manner (0.5 at 0.5 mL h^–1^, then 2.0 at 1.0 mL h^–1^), after which an additional 0.5 mL was added in a single portion. The organic solvent was removed by slow evaporation, and the samples were extensively dialyzed against Milli-Q water (MWCO 100 kDa, regenerated cellulose membrane) for 4 days, with twice-daily medium exchange, to eliminate nonencapsulated enzyme. A free HRP solution subjected to identical dialysis conditions served as control. Purified samples were concentrated using centrifugal filter units (Amicon Ultra-4), and the final HRP content was quantified by UV–vis spectroscopy (NanoDrop 2000c, Thermo Scientific) to determine the encapsulation efficiency.

### Characterization of the Polymersomes

#### Dynamic and Static Light Scattering (DLS/SLS)

Measurements were performed on an ALV/CGS-3 compact goniometer equipped with a 22 mW He–Ne laser (λ = 633 nm), an ALV-7004 digital correlator, and APD single-photon detectors. Samples were placed in 10 mm glass cuvettes and equilibrated at 25 °C. Correlation functions were collected at a scattering angle of 90° with a 30 s acquisition time. Intensity autocorrelation data were analyzed using the CONTIN algorithm to obtain relaxation-time distributions, which were converted into hydrodynamic radius (*R*_H_) by using the Stokes–Einstein equation:

$$R_{H}=\frac{k_{B}Tq^{2}}{6\pi \eta }\tau$$ 1

where *k*_*B*_ is the Boltzmann constant, *T* is the absolute temperature, η is the viscosity of the solvent, and τ is the mean relaxation time. The wavevector (*q*) is defined as *q* = 4π*n*sin­(θ/2)/λ with λ being the wavelength of the incident laser beam, *n* is the refractive index of the solvent, and θ is the scattering angle. The autocorrelation functions were also analyzed using the Cumulant method:^33^

$$\ln g_{1}\left(t\right)=\ln C-\Gamma t+\frac{\mu_{2}}{2}t^{2}$$ 2

*C* denotes the amplitude of the autocorrelation function, Γ the relaxation frequency (τ^–1^), and μ_2_ the second cumulant. Polydispersity indexes were obtained as μ_2_/Γ^2^.

For SLS measurements, the scattering angle (θ) was varied from 30° to 150° in 10° increments. The vesicle molar mass (*M*_*w*(vesicles)_) and radius of gyration (*R*_G_) were extracted from Zimm analysis (eq 3) by using the ALVSTAT software:

$$\left(\frac{Kc}{R_{\theta }}\right)=\frac{1}{M_{w(\mathrm{vesicles})}}\left[1+\frac{R_{G}^{2}q^{2}}{3}\right]$$ 3

where the concentration *c* is given in mg mL^–1^, *K* is the optical constant, *R*_θ_ (Rayleigh ratio) is the normalized scattered intensity (toluene was used as the standard solvent), *n* is the refractive index of the solvent, d*n*/d*c* is the refractive index increment, and *N*_A_ is Avogadro’s number. Therefore, by measuring *Kc*/*R*_θ_ at a given angular range, the value of *R*_G_ is estimated from the slope of the curve, and the molecular weight (*M*_*w*(vesicles)_) is extracted from the linear regression to *q* → 0.

Electrophoretic light scattering (ELS): Surface charge was evaluated by using a Zetasizer Nano ZS (ZEN3600, Malvern Instruments, UK). Measurements were carried out at polymer concentration of 1.0 mg mL^–1^ in 1 mM KCl after 120 s equilibration time. The electrophoretic mobility (*U*_E_) was recorded and converted into ζ-potential values (mV) using Henry’s eq (eq 4):

$$\zeta =\frac{3\eta U_{E}}{2\varepsilon f\left(ka\right)}$$ 4

where ε denotes the dielectric permittivity of the medium and η its viscosity. Henry’s function, *f*(ka), was evaluated using the Smoluchowski approximation, assuming *f*(ka) = 1.5.

#### Cryogenic Transmission Electron Microscopy (Cryo-TEM)

Imaging was performed at the Brazilian Nanotechnology National Laboratory (LNNano) using a JEM 1400 Plus microscope (JEOL) equipped with a LaB_6_ source and operated at 120 kV. A 3.0 μL aliquot was applied to glow-discharged lacey carbon copper grids (300 mesh) and vitrified using a Vitrobot Mark IV (Thermo Fisher Scientific).

#### Small-Angle X-ray Scattering (SAXS)

Small-angle X-ray scattering (SAXS) measurements were conducted at the SWING beamline of the SOLEIL synchrotron facility (Saint-Aubin, France). A wavelength of 1 Å and a sample-to-detector distance of 2 m were used, yielding a scattering wavenumber ranging from 0.04 to 5 nm^–1^. Samples were automatically introduced into a flow-through capillary, and for each of them, approximately 30 two-dimensional scattering images were acquired using an Eiger 4 M detector (Dectris) with a 1-s exposure time per frame, while continuously flowing the sample to minimize radiation damage. The 2D images were converted into *I*(*q*) vs *q* profiles after solvent background subtraction using the FOXTROT software. Subsequent analysis of the SAXS patterns was performed with the SASfit software (Paul Scherrer Institute, Switzerland).^34^

### Evaluation of Polymersome Permeability to Small Molecules

Rhodamine B (RhB) and methylene blue (MB) were used as model probes to assess the membrane permeability to small molecules. Loading efficiency was determined from the ratio between the amount of dye quantified within the vesicles and the initial feed. For quantification, 100 μL of the purified dispersion was mixed with 400 μL of methanol to disrupt the assemblies prior to analysis. Release experiments were performed by placing 6.0 mL of RhB- or MB-loaded polymersomes into cellulose dialysis devices (MWCO 3.5–5.0 kDa) immersed in 1 L of phosphate buffer. At selected time intervals, 100 μL aliquots were collected and diluted with 400 μL of methanol to induce vesicle disassembly before measurement. Quantification of RhB and MB was performed by fluorescence spectroscopy. For RhB, fluorescence emission spectra were recorded from 560 to 620 nm using an excitation wavelength of 520 nm. RhB concentrations were determined by constructing a calibration curve with a linear response in the range from 0.001 to 0.025 μg mL^–1^ prepared under identical medium conditions. For MB, an excitation wavelength of 655 nm was used and emission spectra were recorded from 670 to 800 nm. MB concentrations were quantified using a calibration curve with a linear response in the range from 0.01 to 0.02 μg mL^–1^ also constructed under the same medium conditions.

### Enzymatic Reactions Assays

As for the enzymatic reaction assays, a reaction solution was prepared by adding 200 μL of 10% *v*/*v* H_2_O_2_, 400 μL of acetate buffer (200 mM, pH 5.5), and the appropriate volume of either the HRP-vesicle suspension or free HRP solution to achieve a final enzyme concentration of 1.0 μg mL^–1^. Deionized water was then added to adjust the final volume to 800 μL in a standard UV-vis cuvette. The mixture was used to record the baseline, after which 30 μL of o-dianisidine solution (2.0 mM) was added. The absorption spectra were recorded immediately within the 370–550 nm range with measurements taken every 30 s (Varian Cary 50 spectrophotometer). For polymersome-loaded enzyme, free enzyme, and enzyme-free solutions, time-dependent plots of the maximum absorbance as a function of time were generated.

## Results and Discussion

### Synthesis and Characterization of the Block Copolymers

The block copolymers PGL_15_-*b*-PBO_44_ and PGL_26_-*b*-PBO_78_-*b*-PGL_26_ were synthesized through a tBuP_4_-catalyzed sequential anionic ring-opening polymerization followed by deprotection under acidic conditions.^35^ The synthesis steps are illustrated in Scheme 1.

**Scheme 1:**
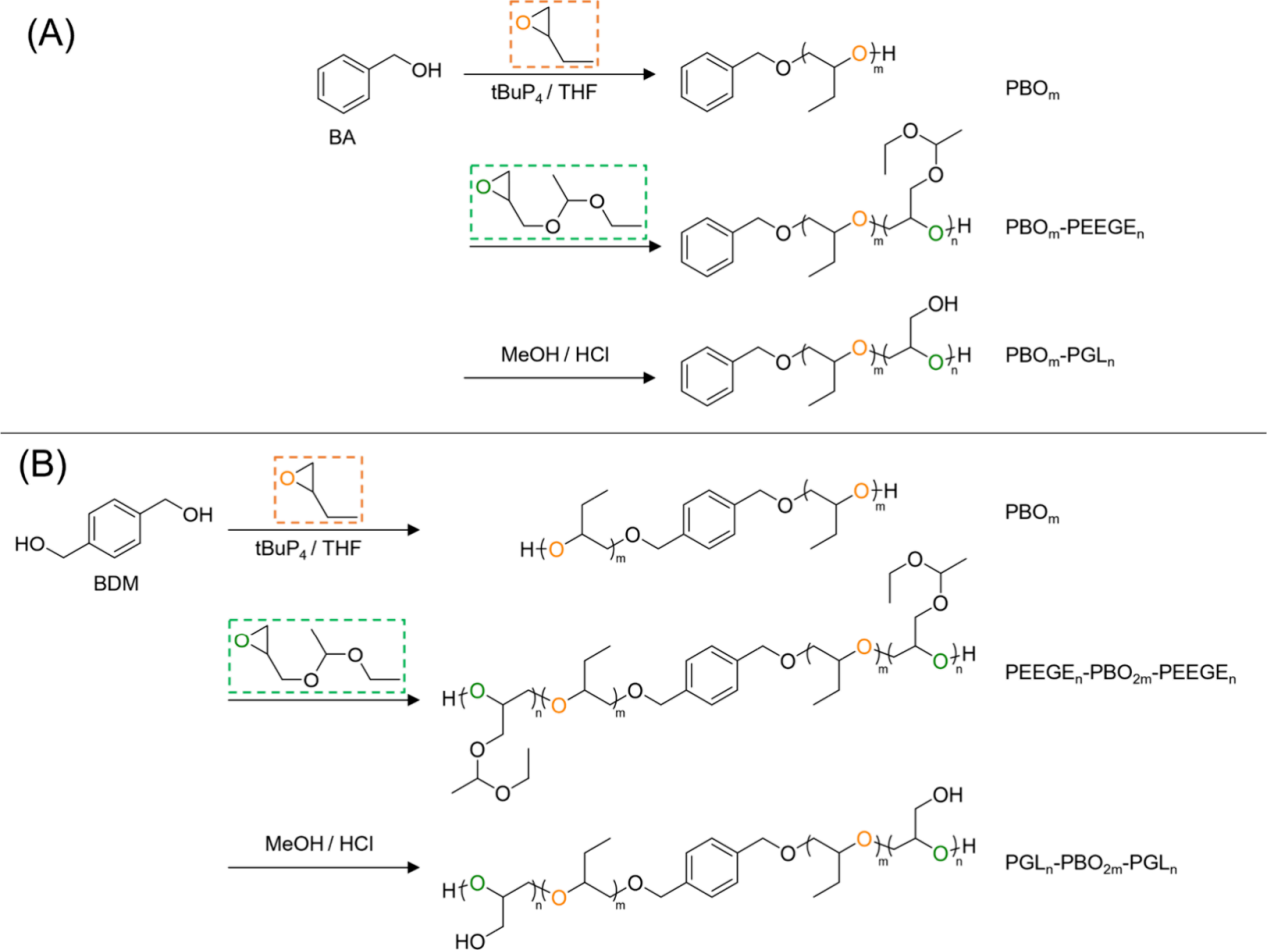
Synthetic Strategy Used to Produce the Linear Block Copolymers PGL_15_-*b*-PBO_44_ (A) and PGL_26_-*b*-PBO_78_-*b*-PGL_26_ (B)

The final polymer structures (Figure 1) and the stepwise evolution during the synthesis were confirmed by ^1^H NMR spectroscopy (Figures S1–S2).

**Figure 1:**
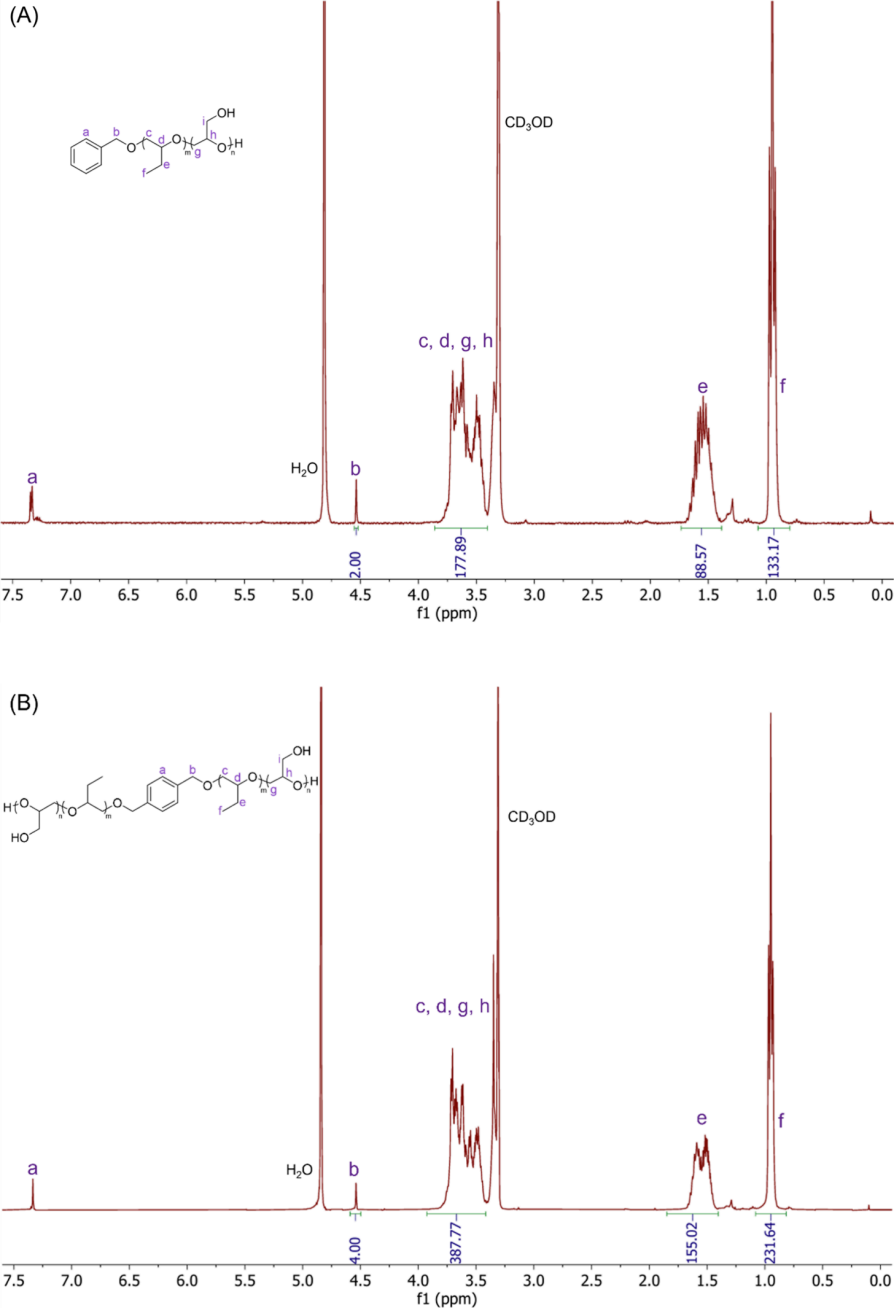
^1^H NMR spectra of PGL_15_-*b*-PBO_44_ (A) and PGL_26_-*b*-PBO_78_-*b*-PGL_26_ (B) block copolymers recorded in CD_3_OD at 400 K.

The characteristic resonances of the aromatic protons of the initiators are clearly visible at δ ≈ 7.3 ppm (a, aromatic protons) and δ ≈ 4.5 ppm (b, −CH_2_–O−). After polymerization of BO, new signals appear at δ ≈ 3.2–3.7 ppm (c, d, backbone −O–CH_2_−) and at δ ≈ 1.5 and 0.9 ppm (e, f, side-chain −CH_2_– and −CH_3_ groups), which are attributed to the repeating units of PBO. These signals confirm the successful formation of the PBO segment, and the respective consumption of BO (Figures S1A and S2A). Upon chain extension with EEGE, additional resonances can be identified as seen in Figures S1B and S2B. The methine proton (j) of the EEGE repeating unit is observed at δ ≈ 4.7 ppm, while the pendant methyl groups give two distinct peaks at δ ≈ 1.3 ppm (k) and 1.2 ppm (m). The methylene protons from the EEGE main chain (g,h,i,l) overlap with those of PBO, resulting in a broad signal centered at δ ≈ 3.2–3.7 ppm. Importantly, the resonance of the catalyst tBuP_4_, visible in the spectrum of PBO (Figures S1A and S2A), disappears after purification, confirming its complete removal. Accordingly, these experimental data underline the successful synthesis of linear block copolymers. Subsequent deprotection of the EEGE units was verified by ^1^H NMR. The characteristic resonances of the acetal protecting groups (j, k, m) completely disappear, while the signals corresponding to the polyglycidol backbone (g, h, i) remain in the δ ≈ 3.2–3.7 ppm region. This demonstrates the efficient removal of the acetal protecting groups and the formation of hydroxy-functionalized PGL blocks.

The degree of polymerization of PBO (m) in the diblock copolymer was calculated from the integral of the methyl protons in the BO unit (proton f) relative to the methylene protons in the initiator (proton b) as m = (*I*_f_/3)/(*I*_b_/2). Similarly, the degree of polymerization of EEGE (n) was calculated from the integral of the methyl protons in the EEGE units (proton m) relative to the methylene protons in the initiator (proton b) as *n* = (*I*_m_/3)/(*I*_b_/2). The final degree of polymerization of the PGL block (n) was derived by comparing the integrals of the protons in the backbone (protons c, d, g, and h) with the methylene protons in the initiator (protons b) as *n* = [(*I*_c,d,g,h_ – *I*_f_)/3]/(I_b_/2). The degree of polymerization of PBO (2m) in the triblock copolymer was calculated using the same integrals with 2m = (*I*_f_/6)/(*I*_b_/4), whereas the degree of polymerization of EEGE (n) was determined from the integral of the methyl protons on the EEGE units (protons k and m) relative to the methylene protons of the initiator (protons b) as *n* = (*I*_k,m_/12)/(*I*_b_/4). The final degree of polymerization of the PGL block was calculated using the same approach as mentioned above with *n* = [(*I*_c,d,g,h_ – *I*_f_)/6]/(I_b_/4). The contribution from the PBO block was subtracted to isolate the signal corresponding to the PGL blocks. These calculations ensured an accurate determination of the PGL degree of polymerization after the deprotection step.

The SEC traces of the block copolymers reveal unimodal distributions (Figure 2) with increased dispersity and tailing observed for the triblock copolymer after acidic deprotection as attributed to side reactions during that last step. The SEC data confirm the successful chain extension and deprotection steps, and they are in good agreement with the ^1^H NMR analyses. Well-defined PGL_15_-*b*-PBO_44_ has been synthesized, while the PGL_26_-*b*-PBO_78_-*b*-PGL_26_ block copolymer structure is in fair agreement with the expected one (Table 1).

**Figure 2:**
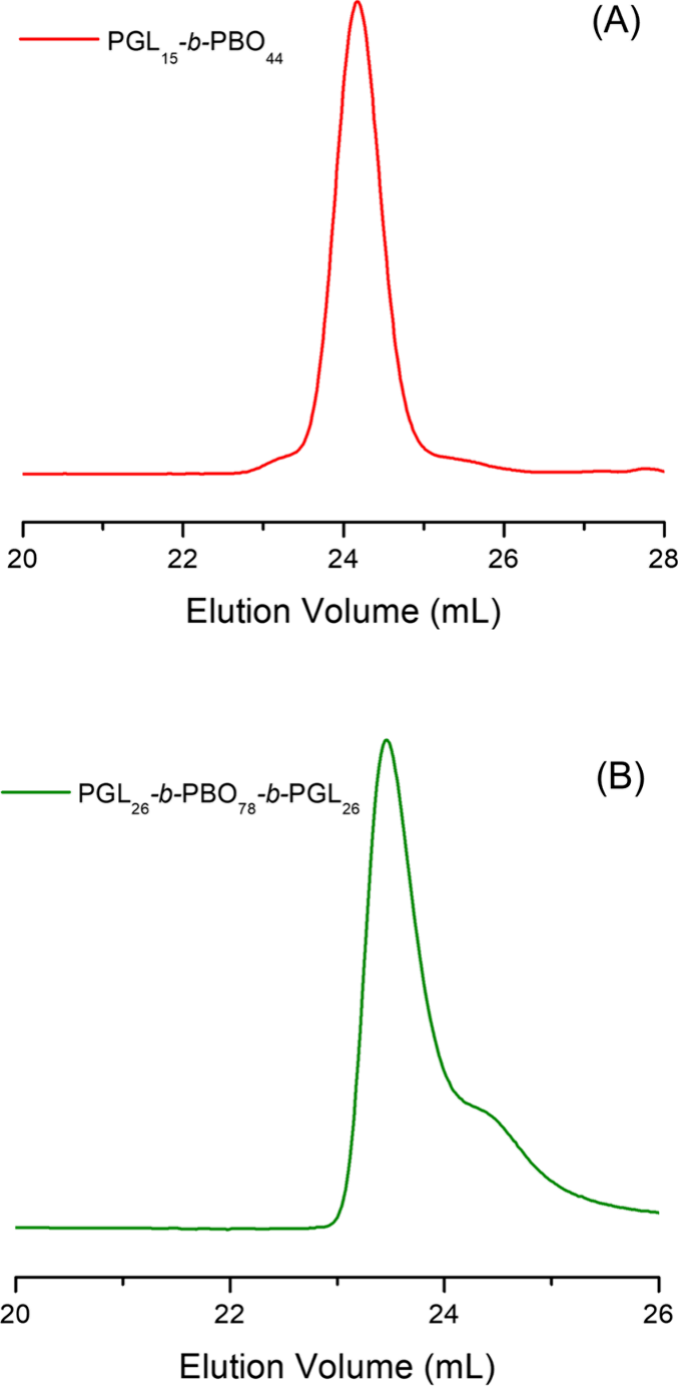
SEC traces for PGL_15_-*b*-PBO_44_ (A) and PGL_26_-*b*-PBO_78_-*b*-PGL_26_ (B) linear block copolymers.

**Table 1:** Structural Characteristics of the Linear Block Copolymers Used in the Investigation

	**^1^H NMR data**		SEC data	
entry	*M*_n(NMR)_ (kg mol^–1^)	*w*_PGL,NMR_	*M*_n(GPC)_ (kg mol^–1^)	*Đ*
PGL_15_-*b*-PBO_44_	4.4	0.25	4.5	1.05
PGL_26_-*b*-PBO_78_-*b*-PGL_26_	9.7	0.39	7.1	1.23

### Production and Characterization of the Polymer Vesicles

The self-assembly of the diblock and triblock copolymers was initially probed by light scattering, as illustrated in Figure 3, and the data are summarized in Table 2.

**Figure 3:**
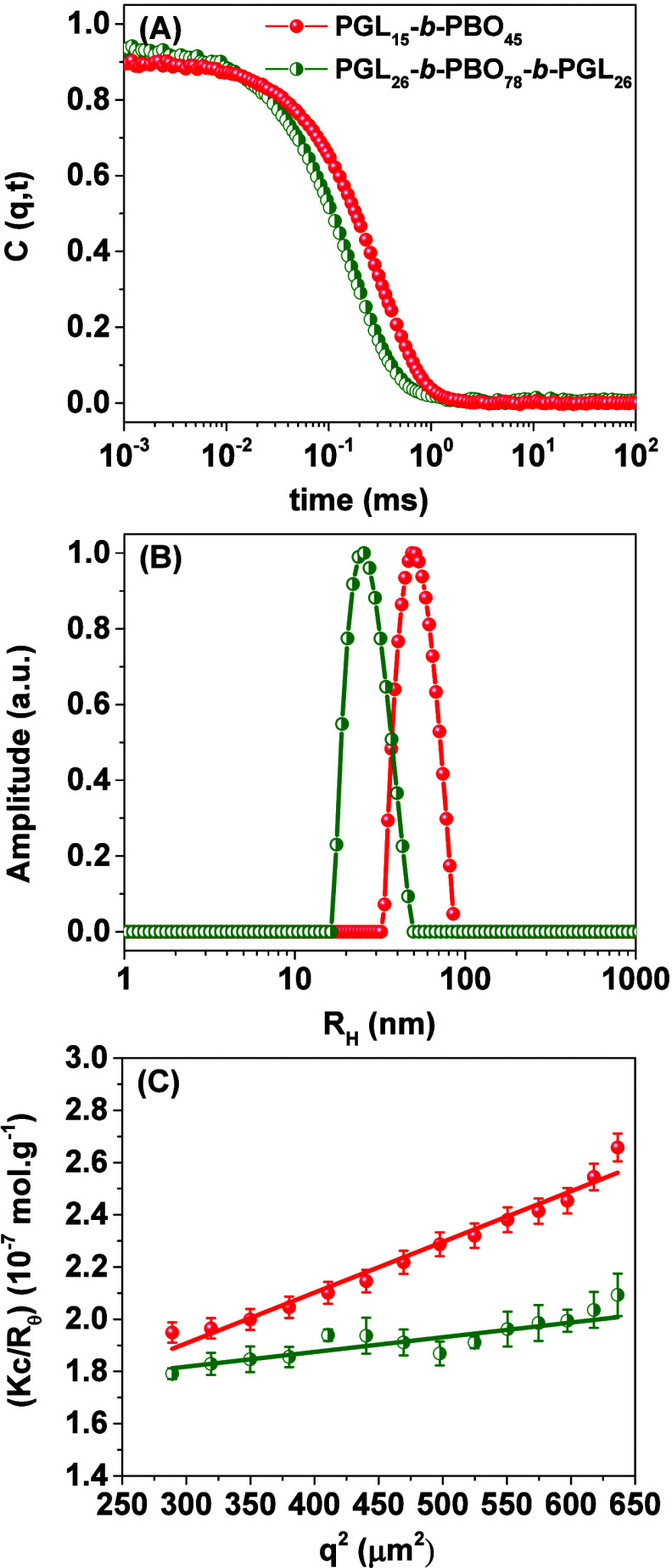
Dynamic and static light scattering characterization of the polymer vesicles: (A) autocorrelation functions, (B) respective distributions of sizes, and (C) Zimm plots for 1.0 mg mL^–1^ polymer solutions (PB, pH 7.4) according to the legend.

**Table 2:** Structural Features of the Manufactured Polymersomes as Determined By Light Scattering

entry	*R*_H_ (nm)	PDI	*R*_G_/*R*_H_	*N*_agg_	ζ(mV)
PGL_15_-*b*-PBO_44_	48.6	0.06	1.17	1600	– 28.2
PGL_26_-*b*-PBO_78_-*b*-PGL_26_	24.1	0.09	1.15	782	–5.1

The dynamic light scattering (DLS) data (Figure 3A,B) indicate the presence of single populations of diffusing particles for both systems, which are nevertheless different concerning the overall size. The triblock copolymer self-assembles in notable smaller vesicles; respectively larger assemblies are reported regarding the diblock copolymer. The average hydrodynamic radii (*R*_H_) were determined to be below 50.0 nm for both block copolymers, yet the radius of the polymer vesicles produced using the diblock copolymer is twice as large compared to that of the polymersomes manufactured using the triblock copolymer. Although the overall size of such assemblies is dependent on the method used in their preparation,^36^ in the present study, the volume of the diblock copolymer vesicles is eight times greater and we further highlight that the reported sizes are highly reproducible between different batches. Static light scattering (Figure 3C) was used to determine the average values of molar mass (*M*_*w*(vesicles)_) yielding relatively high values of resulting numbers of aggregation (*N*_agg_ = *M*_*w*(vesicles)_/*M*_*w*(chains)_). The values are indeed typically obtained for polymer vesicles.^37^ The smaller value (*N*_agg_ = 782) reported for PGL_26_-*b*-PBO_78_-*b*-PGL_26_ is certainly linked to its smaller size, whereas for the diblock copolymer, the value is more than 10^3^. Such notable differences in the self-assembly of the triblock and diblock copolymers deserve further investigations that are currently underway. The polymer library is being expanded in order to investigate in a detailed manner the influence of the polymer configuration, weight ratio (*w*_PGL_), and polymer block lengths in the overall size of the self-assemblies.

Additionally, the radii of gyration (*R*_G_) were derived from the slopes in Figure 3C, and the combination of *R*_G_ (from SLS) and *R*_H_ (from DLS) allows for the determination of the structure sensitive parameter (ρ = *R*_G_/*R*_H_), which is useful to identify different morphologies in the solution. Homogeneous hard spheres have a theoretical value of *R*_G_/*R*_H_ = 0.775, whereas polymersomes are hollow spheres filled with solvent where the amphiphilic membranes concentrate the mass of the objects. Respectively, *R*_G_/*R*_H_ = 1 is assigned for such type of architecture,^37^ which is the case of the herein produced self-assemblies (*R*_G_/*R*_H_ close to 1.0 therefore suggesting the presence of hollow spheres). The self-assembly of the block copolymers into polymer vesicles has been further confirmed by cryo-TEM imaging and SAXS profiles, with respective data provided in Figure 4.

**Figure 4:**
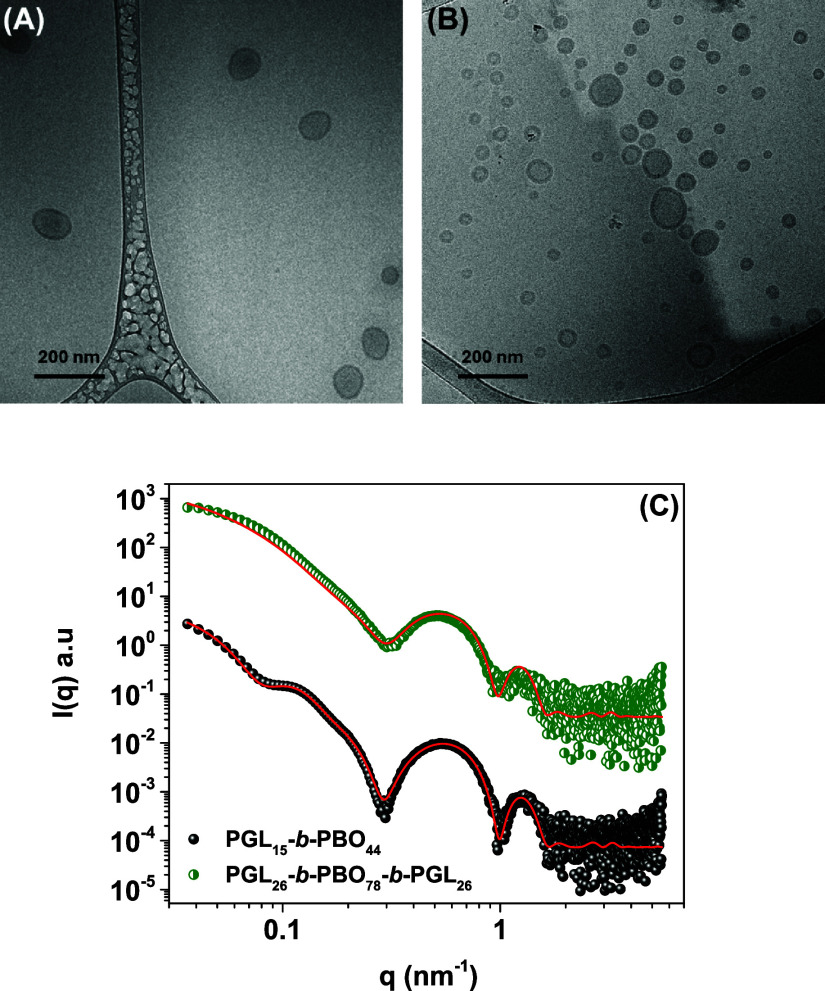
Cryo-TEM images for (A) PGL_15_-*b*-PBO_44_ and (B) PGL_26_-*b*-PBO_78_-*b*-PGL_26_ polymer vesicles produced using a polymer concentration equal to 1.0 mg mL^–1^ in PB (pH 7.4). Small-angle X-ray scattering data (C) with respective curve fitting using the bilayered vesicle form factor for both manufactured block copolymer vesicles at the same concentration and environmental conditions.

The cryo-TEM images show predominantly spherical particles with edge contrast consistent with the presence of polymer vesicles due to the higher electron density of the shell relative to the aqueous lumen, suggesting the presence of hollow morphologies. Size distributions are polydisperse, with some particles significantly larger than others. Yet, clearly smaller vesicles are produced by the self-assembly of the triblock copolymer PGL_26_-*b*-PBO_78_-*b*-PGL_26_. These data are in very good agreement with the previously discussed light scattering results, although the particle size observed by cryo-TEM is commonly smaller than those determined by DLS measurements. Discrepancies are indeed expected because DLS provides an intensity-average size, and thus, cryo-TEM images will usually be undersized relative to DLS data. The size and size dispersity of these assemblies have been frequently checked over at least half a year. They are indeed remarkably stable with no signs of particle aggregation thanks to electrostatic stabilization provided by the negative surfaces (Table 2). We indeed expected that the nanoparticles made from neutral organic polymers would not have neutral surface charge, and therefore, the observation suggests that anions from the aqueous phase preferentially accumulate at the mobile stern layer, and accordingly, negative ζ-potentials are supposed to arise from the accumulation of OH^–^ ions at the interface between water and the organic shell.^38^

The cryo-TEM imaging was complemented by SAXS data, enabling a quantitative assessment of internal dimensions (membrane features). The experimental results reported in Figure 4 underline an upward profile in the low-*q* range, which is typically assigned to relatively large assemblies. The plateau in the low-*q* appears to be already reached for PGL_26_-*b*-PBO_78_-*b*-PGL_26_, thus also pointing out their smaller size compared to that of diblock counterparts, also in very good agreement with light scattering and cryo-TEM images. The absence of more defined features at the low-*q* range confirms the self-assembly of fairly polydisperse hollow spheres, and the oscillations observed in the *q* range ∼0.3–2.0 nm^–1^ are accordingly associated with the membrane characteristics. The scattering profiles could be properly fitted using the bilayered vesicle form factor implemented in the SASfit software as

$$I\left(q\right)={\left(K\left(q,R_{c},\eta_{sol}-n_{h}\right)+K\left(q,R_{c}+t_{h},\eta_{h}-n_{t}\right)+K\left(q,R_{c}+t_{h}+t_{t},\eta_{t}-n_{h}\right)+K\left(q,R_{c}+t_{t}+2t_{h},\eta_{h}-n_{sol}\right)\right)}^{2}$$ 5

with:

$$K(q,R,\Delta \eta )=3V_{\mathrm{sphere}}\Delta \eta \frac{\sin QR-QR\cos QR}{(QR{)}^{3}}$$ 6

The adjustable parameters included the radius of the aqueous lumen (*R*_c_), the thickness of the hydrophilic outer shell (*t*_h_), and the thickness of the hydrophobic inner segment (*t*_t_), along with the respective scattering length densities η, and *V*_sphere_ = (4/3)­π*R* ^3^. The fitted parameters are listed in Table 3.

**Table 3:** Structural Parameters of the Prepared Polymersomes as Determined by SAXS Measurements and Respective Curve Fittings (All Dimensions Are Given in nm)

entry	*R*_*c*_	*t*_*t*_	*t*_*h*_	*w* ^a^
PGL_15_-*b*-PBO_44_	18.2	6.6	3.2	13.0
PGL_26_-*b*-PBO_78_-*b*-PGL_26_	9.0	6.8	2.9	12.6

a Wall thickness (w): t_h_+t_t_+t_h_

The observation of a *q* ^–2^ dependence without distinct low-*q* oscillations indicates notable polydispersity in the core radius (*R*_c_), which was therefore introduced in the fitting procedure. As a consequence, this parameter is associated with significant uncertainty. Modeling with a bilayer form factor yields comparable membrane thicknesses across the different polymer architectures, consistent with the similar positions of the shoulders observed in the SAXS profiles (Figure 4C).

We point out that the *t*_*t*_ value measured presently for PGL_26_-*b*-PBO_78_-*b*-PGL_26_ polymersome fits properly to the produced master curve previously reported by plotting the thickness of the PBO segment (*t_t_*) as a function of the degree of polymerization.^39^ Taking into account that the length of the PBO segment is distinct in the chains (PBO_78_ and PBO_44_), the data suggest distinct configurations within the hydrophobic domains. The repeating unit of PBO is −O–CH_2_–CH­(−CH_2_–CH_3_)–, and the chain unit length has been previously assumed to be around *a*_PBO_ = 0.345 nm.^40^ The thickness of the hydrophobic domain (*t*_*t*_) depends on the degree of polymerization of the PBO segment (*N*), and it scales with *b* = 1 in one boundary (fully stretched chains) whereas *b* = 0.5 is assigned for Gaussian coils, and the value of *b* = 0.66 is expected for entangled chains (*t*_*t*_ = *a*_PBO_.*N* ^*b*^). The value of *t*_*t*_ = 6.6 nm (experimentally obtained) is reached for PGL_15_-*b*-PBO_44_ only when *b* = 0.78 (0.345 × 44^0.78^), which is to some extent unrealistic yet physically possible. Accordingly, this data suggests partial chain interdigitation consistent with a semientangled membrane environment such as reported elsewhere.^40^ As for the case of PGL_26_-*b*-PBO_78_-*b*-PGL_26_, the value of *t*_*t*_ = 6.8 nm is reached for *b* = 0.68 in good agreement with previous investigations reported by us^39^ and by others,^40^ therefore in accordance with the strong segregation theory of block copolymers, indicating strongly stretched chains within the hydrophobic domain of the vesicle membrane. These data also rule out a notable contribution of the low molar mass fraction (Figure 2) in the polymersome formation. Furthermore, stretched chain conformation can also exist due to the moderate hydrophobicity of PBO and respective polymer–water favorable interactions. Last but not least, since this involves the ABA triblock copolymer, the folded configuration of the polymer chains cannot be ruled out and we are currently investigating the self-assembly of a wider polymer library to more robustly understand the reported behavior and chain conformation in the phase-separated systems.

Overall, SAXS data undoubtedly confirm the morphology observed by cryo-TEM and such a combination of light scattering, small-angle X-ray scattering, and cryo-TEM images confirms the self-assembly of the copolymers into polymer vesicles, regardless of the polymer architecture and block length. The experimental data additionally provide relevant insights concerning the internal structure of the self-assemblies.

### Evaluation of the Permeability Behavior

The permeability of the fabricated polymer vesicles toward small molecules was subsequently assessed by using rhodamine B (RhB) and methylene blue (MB) as model hydrophilic fluorescent probes. RhB and MB are both fluorescent organic dyes; however, while RhB contains a xanthene core, MB is a redox-active dye with a phenothiazine core. RhB has strong fluorescence, while MB has a redox-sensitive color and weaker fluorescence. More importantly, MB is permanently cationic, whereas the charges in RhB are balanced out (the carboxylic acid group is deprotonated and the xanthene core is positively charged), making the overall molecule zwitterionic at pH 7.4. The molecular structures of both dyes are provided in Figure 5A.

**Figure 5:**
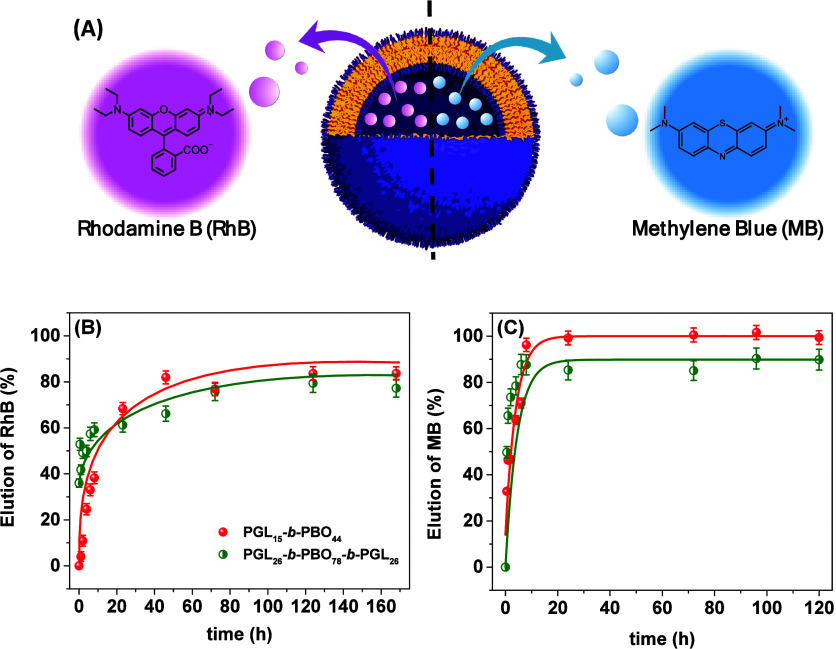
Schematic cartoon of RhB and MB-loaded vesicle release along with the molecular structure of the dyes (A). RhB (B) and MB (C) permeability profiles for different probe-loaded polymer vesicles according to the legend.

During the solvent-switch self-assembly of the block copolymers, RhB and MB are incorporated into the aqueous lumen of the vesicles. The unencapsulated fraction was subsequently removed through gel filtration chromatography, using a Sephadex-based column. The elution profiles of the encapsulated probes were then monitored, and relevant quantitative data are compiled in Table 4.

**Table 4:** Values of RhB and MB Encapsulation Efficiency (EE) as well as Fitted Parameters of Permeability Data Obtained by Adjusting the Experimental Profiles Reported in Figure 5 by Using eq 7, with *CR*_max_ and η Being the Maximum Release and a Characteristic Time, Respectively

block copolymer	EE %	η (h)	*CR*_max_	*R* ^2^	EE %	η (h)	*CR*_max_	*R* ^2^
	rhodamine B				methylene blue			
PGL_15_-*b*-PBO_44_	0.55	10.5	86.78	0.92	0.11	4.0	99.3	0.92
PGL_26_-*b*-PBO_78_-b-PGL_26_	0.12	27.9	77.4	0.87	0.11	4.5	89.8	0.99

First, the experimental data reported in Table 4 underline that the encapsulation efficiency is indeed notably low, regardless of the polymer architecture or the structural features of the self-assemblies. In principle, lower values are expected for the smaller vesicles (PGL_26_-*b*-PBO_78_-*b*-PGL_26_) yet the relatively low values seem to be mostly dictated by polymer chemistry. PBO is a flexible and rubbery polymer at room temperature with glass transition temperature around −60 °C.^41^ PBO has a repeating ether group –O–CH_2_–CH­(−CH_2_–CH_3_)– and, while the overall structure is dominated by nonpolar butylene segments, which do not interact favorably with water, the ether oxygens can interact weakly with water through hydrogen bonding (as an acceptor, not as a donor). Although certainly less hydrophobic, this concept has been explored to explain intrinsic permeable polymer vesicles comprising poly­(propylene oxide) as a hydrophobic segment. The water content has been determined for PPO- (∼23 vol%)^26^ and PBO-containing (∼16 vol%)^27^ block copolymers. Such quantitative data suggest that hydrogen bonding between water molecules and the ether oxygens is indeed likely, thus resulting in water-swollen membranes and also suggesting that the hydration of the hydrophobic domains leads to permeable PBO-based polymersomes, allowing dye diffusion.

We speculate that dye elution occurs even during the purification procedure, resulting in low values of encapsulation efficiency. Even for the polymer vesicles with higher internal volume (those produced using PGL_15_-*b*-PBO_44_), where we expected higher encapsulation efficiency, a similar value has been determined suggesting that indeed EE% is mostly governed by the chemical identity of the vesicles rather than their structural characteristics.

The RhB and MB permeability profiles from the produced loaded polymer vesicles were further monitored, and the experimental data are reported in Figure 5B,C.

The RhB and MB elution trends are to some extent similar regardless of the loaded polymer vesicle where one observes a faster release at initial times with a plateau reached afterward. This behavior is notably distinct compared to 8-hydroxypyrene-1,3,6-trisulfonic acid trisodium salt (HPTS), which has been previously used by us to probe proton permeability in similar vesicles.^39^ This is in principle explained by the highly negative feature of HPTS, which accordingly promotes strong electrostatic repulsions with the negatively charged polymeric shells. This can at least to some extent also be explained by the disparate hydrophilicity of the probes.^28,42^ For instance, Battaglia et al. evidenced that 5,5′-dithiobis-2-nitrobenzoic acid (DTNB), which is more hydrophobic, can permeate PBO hydrophobic membranes, although microvesicles based on poly­(ethylene oxide)-*b*-poly­(butylene oxide) are impermeable to the highly hydrophilic 3,3′,3″-phosphinidynetris-benzenesulfonic acid (PH).^28^ Meier et al. highlighted the formation of impermeable polymer vesicles produced using polyglycidol-*b*-poly­(butylene oxide) when using BODIPY 630/650 as fluorescent probe.^29^ These investigations, along with the current results, thus underline the highly relevant influence of the chemical nature of the diffusant molecule in the reported profiles.

Considering the observed trends, the permeability profiles could decently be fitted by using an exponential growth as

$$\mathrm{CR}(t)={\mathrm{CR}}_{\max}(1-e^{\left(-\frac{t-t_{0}}{\eta }\right)})$$ 7

Here, CR­(*t*) denotes the eluted content as a function of time, while *CR*_max_, η*,* and *t*_0_ correspond to the maximum elution, a characteristic time, and an offset, respectively. The parameter η reflects the permeability kinetics. All fitted values are summarized in Table 4. The release profiles indicate that the polymer vesicles are permeable to the investigated small molecules, as nearly complete probe release is observed over time, consistent with the CR_max_ values determined. Although relying on microreactors, the behavior largely diverges from the evidenced formation of impermeable giant unilamellar vesicles based on PGL_n_-*b*-PBO_m_ block copolymers (BODIPY 630/650 has been used in the mentioned investigation).^29^ Similarly, negligible calcein release has been observed from PEO_n_-*b*-PBO_m_ polymersomes at room temperature (25 °C), which underline once again the key influence of the chemical nature of the loaded molecules.^30^ In this regard, we provide a supporting table (Table S1) summarizing studies concerning dye permeability in PBO-based polymersomes where charge, molecular size, and chemical nature can be compared. Here, we demonstrate a rapid elution with faster release kinetics for MB, as indicated by the η values and visually by the plateau reached before 24 h. This is supposed to be at least to some extent linked to the smaller molecular size of MB and thus faster diffusion across the polymer membranes. Yet, the difference between both probes is mild, suggesting that the distinct charge states of MB (permanently cationic) and RhB (zwitterionic at pH 7.4) do not markedly influence membrane permeability. This observation implies that electrostatic interactions between the dyes and polymer membranes do not play a dominant role in membrane transport. Similar to the characteristic time, the maximum release time is also distinct. The lower plateau reached by RhB is possibly due to its less hydrophilic character compared to MB, thus enabling enhanced hydrophobic interactions with the polymer membrane and finally leading to retention of an RhB fraction within the PBO domains. Interestingly, despite the vesicles exhibiting a negative zeta potential, a condition under which higher encapsulation efficiency for the positively charged MB would be expected, this has not been experimentally observed, strengthening the idea that membrane permeability governs the overall behavior rather than specific electrostatic effects. The determined values of η and EE% do not seem to be correlated with the length of the hydrophobic segment in this molar mass range, or polymer architecture, and the behavior seems to be particularly linked to the chemical features of the hydrophobic block. Yet, investigations with a wider polymer library are underway to clarify these claims.

### Evaluation of the Polymersomes as Nanoreactors

Taking into account the permeable feature of the produced polymer vesicles, as confirmed by the elution profiles of the molecular probes RhB and MB, we have further evaluated their potential as nanoreactors by encapsulating horseradish peroxidase (HRP) into the self-assemblies. Following the quantification of enzyme encapsulation efficiency and purification to remove unloaded HRP, the oxidation of o-dianisidine (o-Di) in the presence of hydrogen peroxide (H_2_O_2_) was carried out. This reaction is catalyzed by peroxidase enzymes such as HRP. The experimental data are listed in Figure 6. Since the enzyme is present only within the lumen of the vesicles after the purification procedure and the reagents are added at the outer compartment (external medium), the product formation can only take place if both H_2_O_2_ and o-Di are able to diffuse across the polymer membranes, thus coming into contact with HRP. This implies that the membranes must exhibit at least a certain degree of permeability to the compounds.

**Figure 6:**
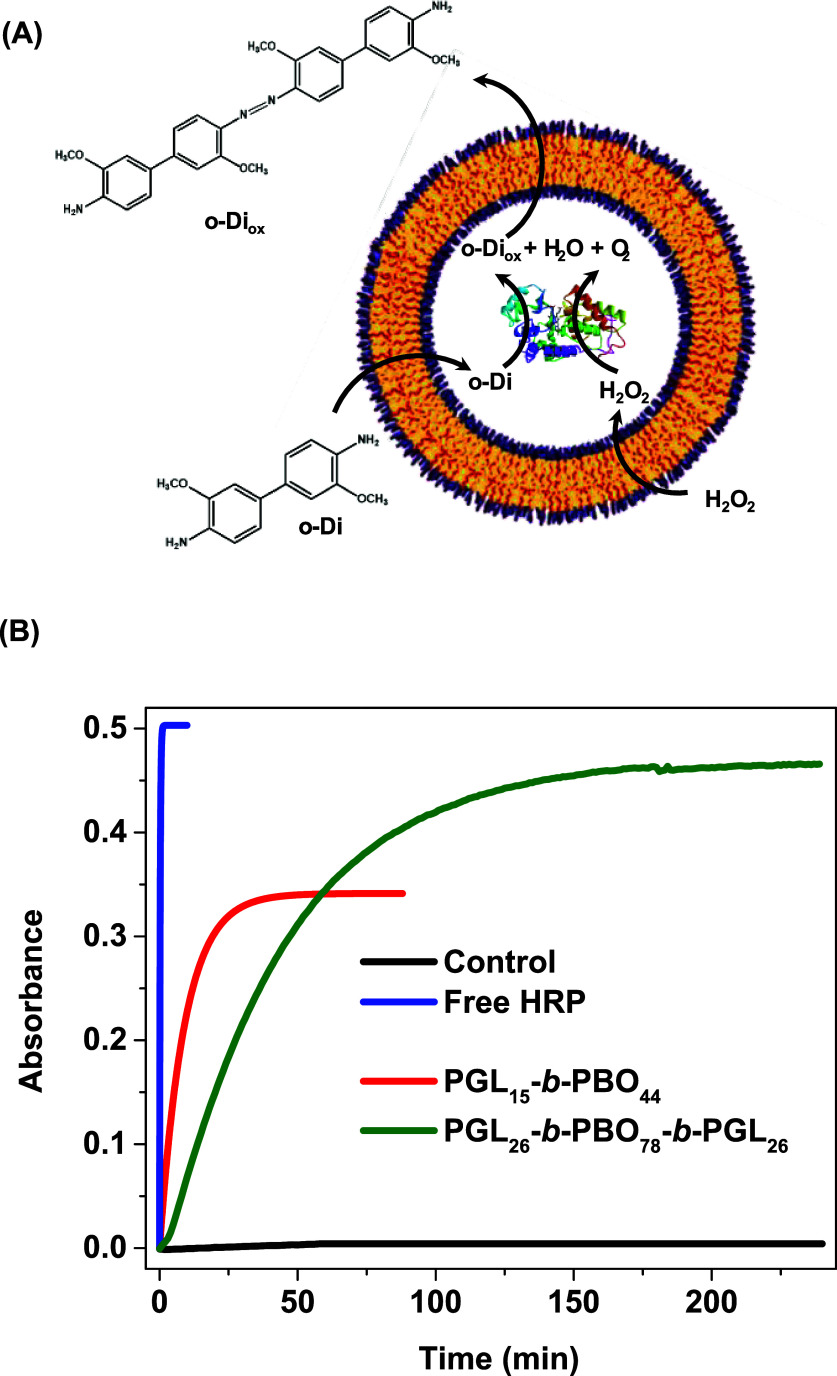
Representation of the enzymatic reaction occurring within the polymersomes (nanoreactors) where the represented protein is HRP, o-Di is o-dianisidine, and o-Di_ox_ is o-dianisidine in the oxidized form (A). Time-resolved UV–vis absorbance traces recorded for the HRP-catalyzed oxidation of *o*-dianisidine (B), with individual profiles corresponding to the different HRP-loaded vesicles, free (nonencapsulated) HRP, and the control sample, as specified in the legend.

Regarding the encapsulation efficiency, values of 41 and 22% were obtained for PGL_15_-*b*-PBO_44_ and PGL_26_-*b*-PBO_78_-*b*-PGL_26_. They are notably higher than the values monitored for the small-molecular probes, which also support the high permeability and leaky feature of such vesicles. The HRP enzyme, on the other hand, is remarkably larger (*M*_*w*_ = 44 kDa), which hampers membrane crossing and release. The different values of HRP encapsulation efficiency are understood to be due to the larger vesicle size produced by using the diblock copolymer (Figure 4).

The o-Di_ox_ formation kinetic inside the vesicles (Figure 6B) exhibits disparate trends. Yet, for both block copolymer vesicles, the absorbance increases over time, clearly evidencing the permeability feature also concerning such reactants. Based on the observed patterns, the permeability profiles could likewise be fitted using the exponential growth model:

$$\mathrm{Abs}(t)={\mathrm{Abs}}_{\max}(1-e^{\left(-\frac{t-t_{0}}{\tau }\right)})$$ 8

Here, Abs­(*t*) represents the time-dependent absorbance, while Ab*s*_max_, τ, and t_0_ denote the maximum absorbance, a characteristic time, and an offset, respectively. The τ values listed in Table 5 provide a qualitative measure of the diffusion rate.

**Table 5:** Values of *R*_H_, HRP Encapsulation Efficiency, and *k* and Ab*s*_max_ for the Produced Polymer Vesicles During Nanoreactor Assay

entry	*R*_H_ (nm)	EE%	τ (min)	Ab*s*_max_
PGL_15_-*b*-PBO_44_	56.0	41	9.6	0.34
PGL_26_-*b*-PBO_78_-*b-*PGL_26_	34.6	22	43.9	0.47
Free HRP			0.22	0.50

The free HRP enzyme exhibits a significantly lower characteristic time constant (τ), as the reaction occurs rapidly due to the immediate contact of odianisidine (o-Di) and H_2_O_2_ with the enzyme in the solution. Conversely, when HRP is encapsulated within the polymersomes, both reactants must diffuse through the vesicular membrane to reach the enzyme and generate the reaction product, retarding the process. The τ values fall within the time scale of minutes for both vesicular systems, and considering that the studies were conducted under conditions aiming to equalize enzyme concentration, they suggest that the permeability of the two vesicles to o-Di and H_2_O_2_ is in the same order. Yet, the value is higher for the triblock copolymer, which suggests an influence of the polymer chain conformation in this time frame. The different plateaus (Ab*s*_max_) are still not clearly understood as various variables have to be considered (characteristic time, volume of aqueous lumen, and particle number, to name a few, are not identical comparing both enzyme-loaded vesicles). We speculate that this is linked to enzyme saturation and consequently inactivation due to markedly fast product formation within the diblock copolymer vesicles. The lower permeability and presumably better flow balance considering incoming reactants and outgoing products in the triblock copolymer assemblies may explain the higher value of maximum absorbance. Nevertheless, this certainly deserves future investigations.

Overall, these results demonstrate that such block copolymers are highly suitable to produce nanoreactors, as they effectively encapsulate sufficient enzyme amounts. Furthermore, the permeable feature of polymer vesicles based on PBO segments is herein robustly confirmed, thus excluding the need of complex or costly membrane permeabilization methods to allow the flow of reaction substates.

## Conclusions

In the present investigation, and following early studies where the permeability of PBO-based vesicles has been suggested,^28^ we have optimized the synthesis of diblock and triblock copolymers comprising hydrophobic PBO and hydrophilic PGL segments. Furthermore, the self-assembly behavior, and particularly the feasibility to produce polymer vesicles, was probed using scattering and imaging approaches, which have indeed robustly confirmed the desired morphology. The polymer vesicles were notably permeable to small molecules despite differences in the chemical structure of the probes or the conformation and stretching state of PBO segments, thus suggesting that the behavior is mostly governed by the chemical structure of the membrane rather than their thickness or other structural characteristics. These data revealed the manufacturing of permeable nanoreactors without the aid of further external agents, which was indeed proved afterward. The PBO layer enables confinement of large macromolecules, such as enzymes, while its intrinsic permeability to small molecules underscores the suitability of this polymeric material for creating confined reaction spaces applicable to a broad range of enzymes for biomimetic and nanomedicine contexts. We are extending this investigation by synthesizing a broader library of block copolymers, which will allow a systematic evaluation on the influence of the polymer architecture (diblock vs triblock copolymers), membrane thicknesses, and polymer conformation on permeation rates. Furthermore, different methodologies will also be used toward the production of giant vesicles (microreactors) that are leaky by design.

## Supplementary Material



## Notes

^1^H NMR spectra of PBO and PEEGE_n_-*b*-PBO_m_ during the synthesis of PGL_15_-*b*-PBO_44_ and of PBO and PEEGE_n_-*b*-PBO_2m_-*b*-PEEGE_n_ during the synthesis of PGL_26_-*b*-PBO_78_-*b*-PGL_26_; table with specific probes employed to investigate the membrane permeability of PBO-based polymer vesicles (PDF)
