# Supplementary material for: Leaky by Design: Unlocking Polymersome Permeability Using Moderately Hydrophobic Polymer Blocks
Source: Langmuir. 2026 Mar 11;42(11):7872–84. doi: 10.1021/acs.langmuir.5c06389 (PMC13019673; doi:10.1021/acs.langmuir.5c06389)
Supplement: Supplementary file 1 [file la5c06389_si_001.pdf]

Supporting Information File

Leaky by Design: Unlocking Polymersome Permeability Using Moderately Hydrophobic Polymer Blocks

Wencui Zhang,[1♠] Anabella P. Rosso,[2♠] Yang Yu,[1] Fernando Augusto de Oliveira,[2] Martina Vragovic,[3] Cécile Huin,[1,4] Philippe Guégan,[1] Guillaume Tresset,[5] and Fernando Carlos Giacomelli[1,]*

[1] Equipe Chimie des Polymères, Institut Parisien de Chimie Moléculaire (UMR-CNRS 8232), Sorbonne Université, 75 252 Paris, France

[2] Centro de Ciências Naturais e Humanas, Universidade Federal do ABC, 09210-580 Santo André, Brazil

[3] Institute of Macromolecular Chemistry, Czech Academy of Sciences, 162 00 Prague, Czech Republic

[4] Université Evry Paris-Saclay, 91 000 Evry, France

[5] Université Paris-Saclay, CNRS, Laboratoire de Physique des Solides, 91 405 Orsay, France

♠*These authors contributed equally to this work.*

*Corresponding Author:

Fernando Carlos Giacomelli: fernando.giacomelli@ufabc.edu.br

Supporting Information Content:

Number of pages: 4

Number of figures: 2

Number of schemes: 0

Number of tables: 1

List of the content in Supporting Information:

^{1}H NMR spectra of PBO and $PEEGE_n$-*b*-PBO_m during the synthesis of PGL_{15}-*b*-PBO_{44} (Figure S1) and of PBO and $PEEGE_n$-*b*-PBO_{2m}-*b*-$PEEGE_n$ during the synthesis of PGL_{26}-*b*-PBO_{78}-*b*-PGL_{26} (Figure S2). Table with specific probes employed to investigate the membrane permeability of PBO-based polymer vesicles (Table S1).

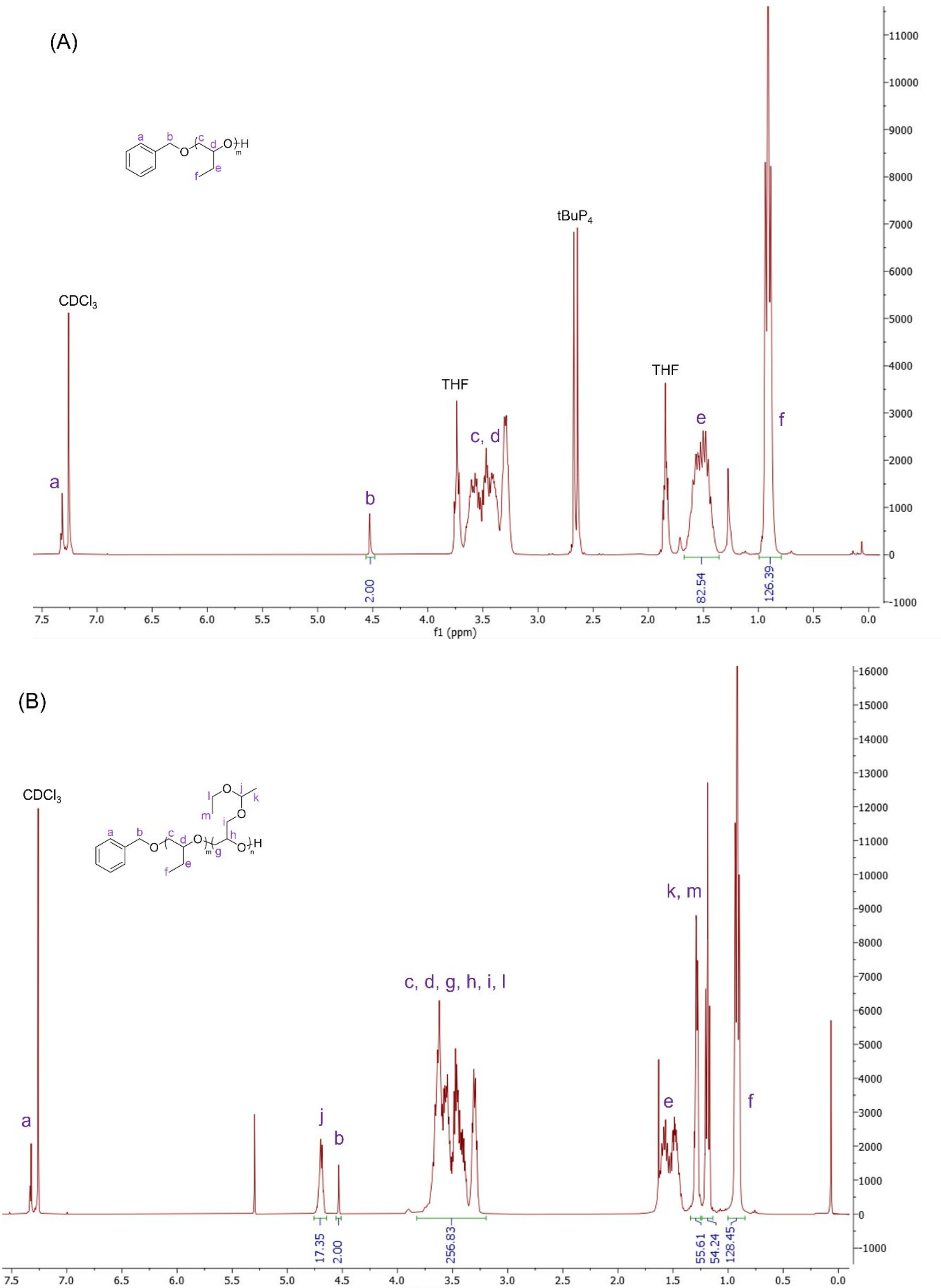

Figure S1. ^{1}H NMR spectrum of PBO (A) and PEEGE$_n$-*b*-PBO$_m$ (B) during the synthesis of PGL$_{15}$-*b*-PBO$_{44}$. The polymers were dissolved in deuterated chloroform ($CDCl_3$) and the spectra were acquired at room temperature.

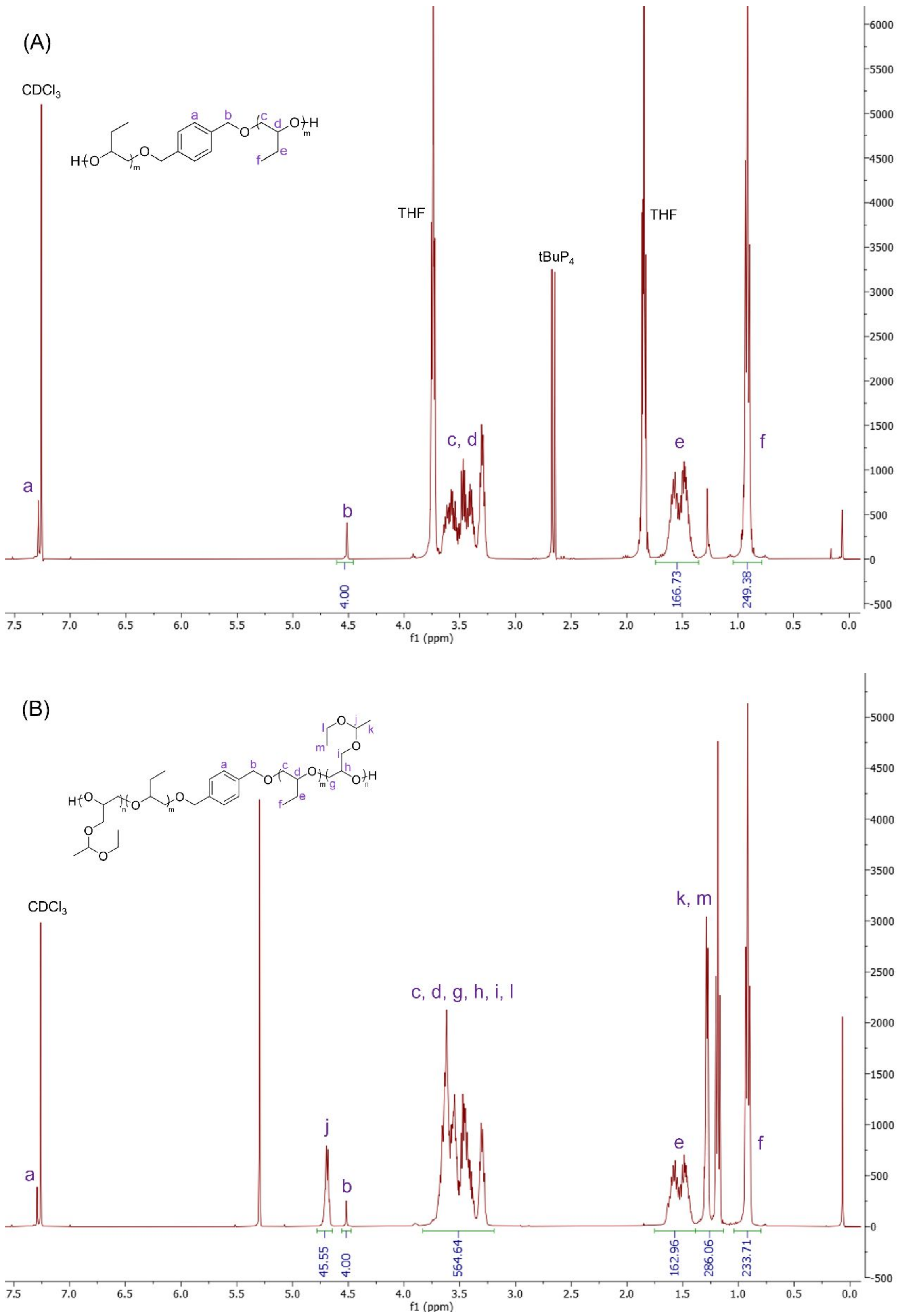

Figure S2. ^{1}H NMR spectrum of PBO (A) and PEEGE$_n$-*b*-PBO$_{2m}$-*b*-PEEGE$_n$ (B) during the synthesis of PGL$_{26}$-*b*-PBO$_{78}$-*b*-PGL$_{26}$. The polymers were dissolved in deuterated chloroform ($CDCl_3$) and the spectra were acquired at room temperature.

Table S1. Specific probes employed to investigate the membrane permeability of PBO-based polymer vesicles.[a]

Probe	Probe Structure	Molecular Weight	Charge at pH ~ 7	Permeable	Ref
5,5′-dithiobis-2-nitrobenzoic acid	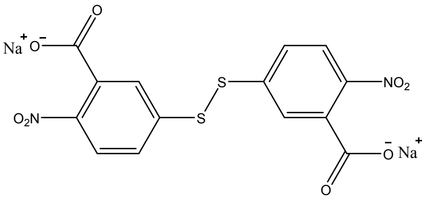	396.4 $g.mol^{-1}$	Negative	✓	28
3,3′,3″-phosphinidynetris-benzenesulfonic acid (salt form)	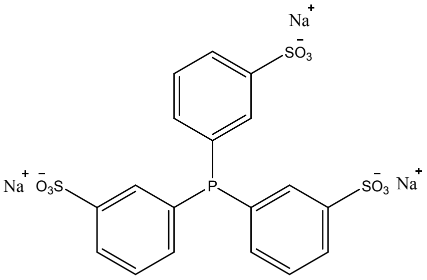	568.4 $g.mol^{-1}$	Negative	✗	28
BODIPY 630/650	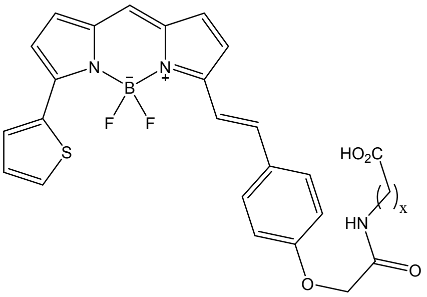	~ 600 $g.mol^{-1}$	Neutral	✗	29
Calcein	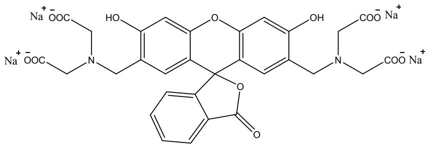	622.5 $g.mol^{-1}$	Negative	✗	30

8-hydroxypyrene-1,3,6-trisulfonic acid trisodium salt	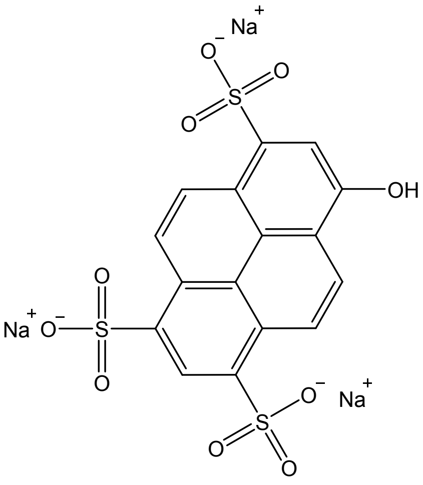	524.4 $g.mol^{-1}$	Negative	✗	39
Rhodamine B	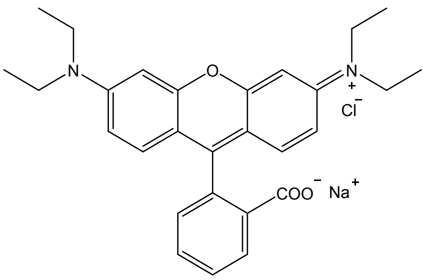	479.0 $g.mol^{-1}$	Neutral	✓	*
Methylene blue	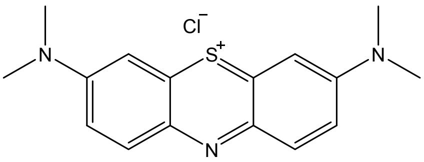	319.8 $g.mol^{-1}$	Positive	✓	*

[a] readers are suggested to go through the mentioned references for details of the probes as several derivatives may exist.

* this investigation
